# Dry Lithography of Large-Area, Thin-Film Organic Semiconductors Using Frozen CO_2_ Resists

**DOI:** 10.1002/adma.201202446

**Published:** 2012-09-11

**Authors:** Matthias E Bahlke, Hiroshi A Mendoza, Daniel T Ashall, Allen S Yin, Marc A Baldo

**Affiliations:** Department of Electrical Engineering and Computer Science, Massachusetts Institute of TechnologyCambridge, MA 02139, USA E-mail: mbahlke@mit.edu; School of Electronic Engineering, Bangor UniversityDean Street, Bangor, LL57 1UT, UK

**Keywords:** patterning, organic, semiconductors, OLED, lithography

The molecular constituents of organic light emitting devices (OLEDs) are typically soluble in organic solvents. Consequently, OLEDs cannot be exposed to the key components of conventional photolithographic processes. The OLED industry relies instead on thermally evaporated thin organic films that are patterned by metal shadow masks. But shadow masks presently limit OLED displays to smaller substrate sizes than their liquid crystal display (LCD) counterparts.[1–3] Serial printing techniques may eventually provide a solution if they can be sufficiently parallelized to reduce takt time,[4–9] the cycle time of the process. The ultimate goal for OLED manufacturing, however, is to replicate the widespread success of photoresist lithography. Hence, there is motivation for a renewed examination of variants of this inherently parallel, high speed approach.

Traditional photolithographic techniques have been applied to organics by making use of their orthogonality with highly flourous materials.[10–12] They've been patterned by crosslinking and dissolving away undesired regions of the organics themselves akin to photoresists.[13] A polymer film has been shown to protect organic materials during standard photolithography steps.[14] In addition, super critical carbon dioxide has been employed to dissolve resists without interfering with the active material.[15] Despite demonstrating respectable figures of merit, shadow masking remains the industry standard.

As early as 1978, IBM researchers investigated dry lithographic patterning of thin films using sublimation of a phase-change resist.[16], [17] The absence of solvents, or liquids of any kind, may make such a process compatible with OLED patterning. More recently, Golovchenko et al. have used frozen H_2_O resists to pattern features at the nanometer scale.[18–20] But water has been shown to cause degradation processes in OLEDs.[21], [22] Instead, we consider the application of inert, frozen carbon dioxide (CO_2_) to the lithography of OLEDs.

A typical process flow for phase-change lithography is shown in **Figure** **1**. The resist gas is first applied to a cryogenically cooled substrate where it freezes. The desired pattern can be formed in the resulting resist film via localized thermal excitation; in this work, we investigate resistive heating and stamping. As explained below, stamping is preferred, and this process of pattern transfer is described in Figure 1c. Following physical vapor deposition of the organic semiconductor or metal, the substrate temperature is raised and the resist sublimes, lifting off unwanted materials, and leaving behind only the intended pattern of organic or metallic thin film. The material that is lifted off can be caught by a shutter below the sample or, in the case of a manufacturing line, the step can be performed in a separate chamber to recover the material for reuse. A video of the frozen CO_2_ mask lifting off is available in the Supporting Information.

**Figure 1:**
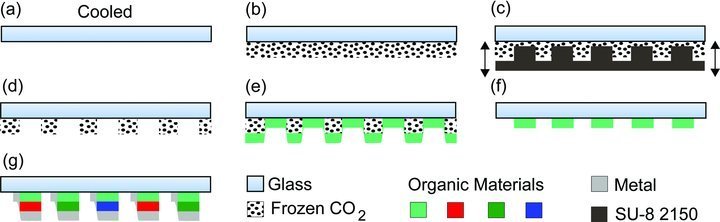
Simplified process flow for sublimation lithography using a frozen CO_2_ resist (not to scale). (a) Cool substrate below 100 K. (b) Freeze on thin film of CO_2_. (c,d) Pattern CO_2_ film by heating selectively. Here a stamp is pressed against the resist to remove some areas. (e) Deposit desired organic or metal thin film by thermal evaporation. (f) Warm substrate to sublime CO_2_, thus lifting off unwanted material. (g) Repeat steps (a–f) as necessary to complete the device.

To avoid degradation of our active materials, we employ inert CO_2_ as the phase-change resist. The phase diagram of CO_2_ is shown in **Figure** **2**a.[23] In a low pressure process like thermal evaporation, the sublimation temperature of CO_2_ is reduced. For example, at our operating pressure of 10^−6^ Torr, the sublimation temperature of CO_2_ is roughly 90 K. Thus, for a stable lift-off mask, the substrate must be cooled to at least 85 K, which is still within the range of relatively inexpensive cooling with liquid nitrogen.

**Figure 2:**
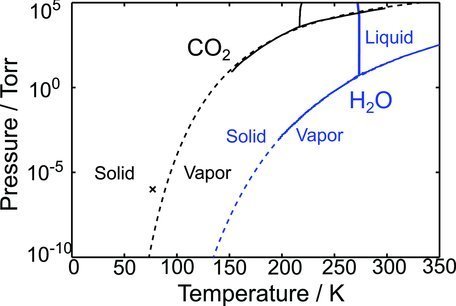
Phase diagrams of CO_2_ and H_2_O extrapolated (broken lines) from data (solid lines) in Refs [23] and [24]. The “×” represents the process operating point of 77 K at 10^−6^ Torr. The curves are extrapolated using the Clapeyron equation.[33]

Once the CO_2_ resist is patterned by selective sublimation, it is important to control the partial pressure of CO_2_ in the chamber to prevent unwanted re-condensation of CO_2_ vapor on patterned regions of the substrate. It is also possible to freeze other impurity gases onto the substrate, notably H_2_O, whose phase diagram is shown in Figure 2b.[24] In previous studies of frozen CO_2_ films at 10^−7^ Torr, Gerakines et al. measured a water deposition rate of 2 nm h^−1^.[25] At these rates re-deposition must be considered in our experiments, but should ultimately be of little consequence in high throughput manufacturing since the acceptable background pressures of CO_2_ and H_2_O increase with reduced takt time.

The ultimate resolution of this lithographic process is determined by the resist thickness, which in turn is limited by the thickness of solid CO_2_ required to withstand the thermal energy carried by the incident organic or metal film.[26] To estimate the minimum resist thickness, *t_CO_*_2_, of an unpatterned film, we consider the balance of the heat capacities *c_v_*, enthalpy of sublimation *h_s_*, and heats of fusion *h_f_* and vaporization *h_v_* of the resist and evaporated materials:


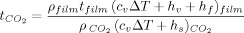
(1)

where *ρ* and *t* are the density and thickness of a material, respectively. The subscripts *film* and *CO*_2_ correspond to the evaporated thin film to be patterned and the resist, respectively. The minimum resist thickness obtained using Equation 1 is approximately 3 μm and 400 nm for 100 nm of deposited silver and standard organic materials, respectively. The density of the resist depends on pressure, temperature,[27] and gas flow rate and, as mentioned in reference [28] a more amorphous resist avoids inhomogeneity at the length scales of the crystalline domains and is preferred for greater resolution. For the operating conditions in these experiments, the density is 1.51 ± 0.15 g cm^−3^; see the Supporting Information for a description of the interferometric technique employed to measure density and film growth rates.

The inherent disadvantage of phase-change lithography complicating the patterning step is the relatively large amount of thermal energy that must be supplied to overcome the heat of sublimation and remove the resist during patterning.[18] Traditional optical lithographic exposure methods would require a great deal of power at wavelengths that are not readily available, λ = 2.7 or 4.3 μm,[29] to achieve a suitable sublimation dose in a reasonable amount of time. Thus, although a number of selective heat sources are possible, we investigated resistive heating and a stamping technique to selectively sublime regions of the CO_2_ mask. Both techniques are capable of rapidly injecting a significant amount of heat into the CO_2_ resist.

Resistive heating was performed by applying a voltage along an indium tin oxide wire patterned on a glass substrate. At a current density of 625 kA cm^−2^, the heat dissipated by the resistive load is sufficient to sublime the overlying frozen CO_2_. An in vacuo photograph of this arrangement is shown in **Figure** **3**a.

**Figure 3:**
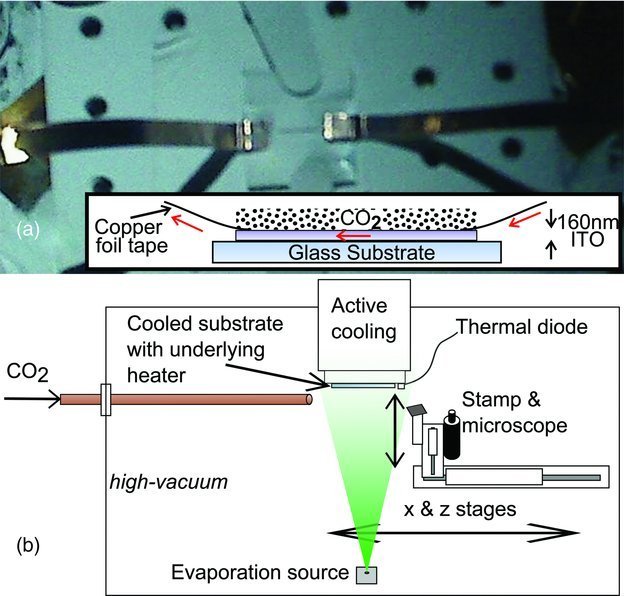
Optical micrograph and schematic representation (a) of the resistive heating method of patterning the frozen mask. Electrical contacts on the substrate allow current to be driven at a current density of 625 kA cm^−2^ through a 160 nm-thick strip of ITO on the substrate. The red arrows in the schematic drawing indicate the flow of current through the ITO enabled by affixed copper foil tape. Schematic of the experimental setup (b) showing the process-critical components as described in the main text. The entire setup is located within a standard thermal evaporation chamber (Angstrom Engineering). The arrows by the stages indicate their respective direction and range of motion. Both the stamp and the microscope used for positioning and stamping evaluation are located on the smaller stage as indicated.

A schematic representation of the setup used for pattern transfer by stamping is shown in Figure 3b. The process is performed using two motorized linear stages (Standa Ltd.) to allow for motion control under vacuum: a 150 mm motorized linear stage to traverse the length of the cryogenically cooled sample and another, 30 mm in length, with actuation normal to the substrate to perform the stamping operation and to adjust the focus of a digital microscope. The microscope is used for positioning the stamp relative to the substrate and to observe the sample surface throughout the process.

In a demonstration of patterning after resistive heating, an approximately 100 μm-wide silver line is patterned by this method; see **Figure** **4**a. The silver wire is observed to follow the outline of the underlying indium tin oxide (ITO) wire where it is narrowest, and the heat generation is the largest. Due to the high heat flux and diffusion through the substrate, however, this method yields an insufficiently rapid sublimation process resulting in cruder definition and undesirable debris. For this reason, the method was abandoned in favor of the stamping technique that heats the resist rapidly and directly, rather than through the substrate.

**Figure 4:**
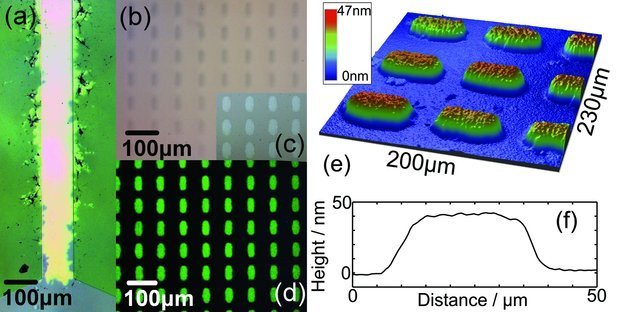
Micrograph (a) of a 100 μm-wide thin film of silver patterned using a CO_2_ mask. The mask itself is defined by running 100 mA (625 kA cm^−2^) of current through a strip of a 160 nm-thick transparent conducting oxide that is also visible beneath the metal film. Optical micrograph (b) of a 78 μm pitch-patterned mask of CO_2_ defined via contact with a stamp. The corresponding inset (c) shows the Alq_3_ thin film after deposition and lift-off. Photoluminescence micrograph (d) from the same film. A false-color topography obtained by an optical interferometer (e) and a cross section of the same (f) detail the profile of the patterned organic pixels.

To demonstrate pattern transfer using a stamp, arrays of 20 μm × 50 μm features were patterned from thin films of the common organic compound tris(8-hydroxyquinolinato) aluminum (Alq_3_). A micrograph of one of these stamps is available in the Supporting Information. This resolution is compatible with commercial OLED display production, and exceeds that which might be used in a mobile display. The CO_2_ mask and the subsequent patterned Alq_3_ thin film are shown in Figures 4b and c. Below that Figure 4d is a photoluminescence micrograph of a λ = 405 nm LED-pumped array of Alq_3_ pixels with 78 μm pitch. For clarity, a longpass wavelength filter was used to remove the pump from the image. Profiles of patterned pixels were acquired with an optical profiler (Veeco Instruments Inc.) and contact surface profilometers (Veeco, Tencor). Figure 4e is a false-color surface topography showing multiple pixels while Figure 4f shows the two-dimensional cross section. The relative heat capacity of a stamp maintained at room temperature is more than sufficient to rapidly remove the frozen resist. To prevent abrasion and dust formation, the surface of the stamp need not make contact with the hard substrate surface if a universal burn-off step is performed to uniformly ‘etch’ the residual resist.[19] In this step, all of the resist is uniformly removed a suitable depth such that none remains in the areas where the desired thermally deposited film is to remain.

To examine the impact of cold substrate temperatures on OLEDs, we built and tested OLEDs on substrates cooled to 112 ± 24 K. The external quantum efficiency (EQE) versus current density, *J*, and electroluminescence spectrum of these devices are shown in **Figure** **5**. The best devices on cold substrates yielded efficiencies comparable to the room temperature grown control devices, suggesting that cooled substrate temperatures can be employed in OLED fabrication without degradation of performance. The performance and yield of the cold OLEDs was highly variable, however, and we observed a visible grey tint in the hole transport layer due to a slight coarsening in the morphology. While morphological changes might occur during low temperature depositions, we attribute the significant variation in device performance to the uncontrolled condensation of water vapor or CO_2_ on our substrate surface during substrate cooling and the growth of the thin films.[21], [22] This can be rectified by reducing the takt time from the ∼1 h process used in our laboratory, and reducing the partial pressures of water and CO_2_ using cold traps.[28]

**Figure 5:**
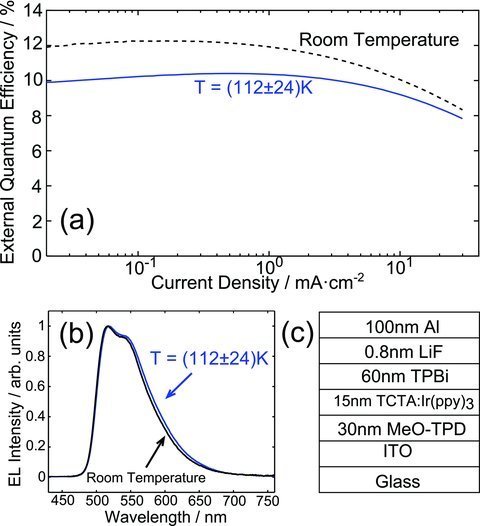
External quantum efficiency versus current density of OLEDs grown at T = 112 ± 24 K and room temperature (a). The normalized electroluminescence spectrum is indistinguishable from the room temperature control device (b) and device thin film stack (c) are also shown.

In addition to addressing concerns of the organic layers' growth under cold conditions, the transistor backplane of active-matrix displays must also withstand the low temperatures of the process. To verify this, a small active-matrix OLED display was removed from a digital photo frame, pumped down to high vacuum and cooled with liquid nitrogen, and then replaced in its housing and connected to its driver. There was no noticeable difference in pixel brightness, uniformity or operation aside from the seal of the passivation glass coming loose—passivation being a manufacturing step strictly after full device fabrication.

To conclude, the use of an in situ definable mask has been shown to be a viable alternative to patterning thin films of organic semiconductors and metals at the large scale. The dry resist material frozen directly to the surface of the substrate alleviates many of the issues of scaling up as fine metal masks have proven increasingly cumbersome with area. While pixel density as high as 325 pixels-per-inch has been demonstrated, there is no reason to believe this is a fundamental limit—especially knowing that nanometer-scale patterning has been demonstrated with an electron beam paired with frozen water resist.[18–20] Patterning organics at the nanoscale may be possible pairing phase-change resists with nanoimprinting techniques. Employing a micro-featured stamp roller pipelined with the necessary cooling apparatuses, phase-change resist patterning should allow for scaling of parallel patterning beyond what the current technologies offer.

## Experimental Section

Patterning was successfully demonstrated within a vacuum in the 10^−7^ to 10^−6^ Torr range for substrate temperatures between 20 K and 100 K. Cooling is achieved using either a liquid nitrogen reservoir or a repurposed cryogenic pump depending on the desired base temperature. With the substrate sufficiently cooled, CO_2_ gas (Airgas, 99.999%) is introduced to the substrate via 1/4” copper tube attached to either a mass flow controller or variable leak valve depending on the desired flow rate; the flow rate also being a function of temperature and pressure; see Supporting Information.

In the resistive heating experiments, 160 nm-thick ITO was patterned by traditional contact photolithography and etched with aqua regia. Copper foil tape was used to make contact from the ITO on the substrate to a ceramic power feedthrough. A sourcemeter (Keithley 2400) was used to drive 100 mA of current through 100 μm-wide lines resulting in heating at a rate of 225 mJ cm^−2^ s^−1^ until the frozen CO_2_ formed the intended pattern observed from a camera mounted in situ.

We used SU-8 2150 photoresist to fabricate our stamps following prior reports.[30] When spun onto silicon wafers at 3,000 RPM, thicknesses of ∼115 μm were obtained. Contact photolithography resolved pillars that tapered slightly after development in propylene glycol methyl ether acetate (PGMEA). For these experiments, the tapering is not so severe as to interfere with patterning as the resist thickness is on the order of 50 μm. The thickness and density of frozen CO_2_ films is measured by double interferometry, as detailed in the Supporting Information.[27], [31], [32]

For experiments with temperatures less than the ∼80 K obtainable with a liquid nitrogen reservoir, a cryogenic pump was repurposed for use as a cooling source and all components are mounted onto it via an oxygen-free high-conductivity (OFHC) copper rod. All cold parts are machined out of OFHC copper and indium foil is sandwiched between all temperature-critical interfaces. A kapton encapsulated heater placed in between the substrate and substrate holder provides adequate local heating for encouraging lift-off without adding too much heat to the bulk thermal mass of the apparatus. A silicon thermal diode was attached to the copper substrate holder to approximately monitor the temperature of the sample and a cryogenic temperature controller (Lakeshore Cryotronics) is employed to manage operating temperature. Patterning is monitored using a digital microscope mounted on the stamp actuator. The repurposed cryogenic pump's compressor and cold head are briefly turned off during the actual stamping so that the vibrations do not interfere while the stamp and resist make contact.

We fabricated OLEDs using standard shadow-masking techniques to demonstrate compatibility with the low temperature substrates. Basic phosphorescent OLEDs employing fac tris(2-phenylpyridine) iridium (Ir(ppy)_3_) as the emitter were grown on ITO-coated glass substrates at similar temperatures as the stamping process, but active areas were defined using traditional shadow-masking techniques so as to evaluate the critical temperature-dependent parameter of using CO_2_ as a lift-off resist in producing organic optoelectronic devices. The substrates were detergent, solvent, and plasma cleaned prior to device fabrication. No CO_2_ was introduced in these experiments. *N,N,N′,N′*-tetrakis(4-methoxyphenyl)-benzidine (MeO-TPD), 4,4′,4″-tris(carbazol-9-yl)triphenylamine (TCTA), 2,2′,2″-(1,3,5-benzinetriyl)-tris(1-phenyl-1-H-benzimidazole) (TPBi), and lithium fluoride (LiF)/aluminum (Al) were used as the hole transport, host, electron transport and cathode layers respectively.

## Supplementary material

Detailed facts of importance to specialist readers are published as ”Supporting Information”. Such documents are peer-reviewed, but not copy-edited or typeset. They are made available as submitted by the authors.
