# Supplementary material for: Dry Lithography of Large-Area, Thin-Film Organic Semiconductors Using Frozen CO2 Resists
Source: Adv Mater. 2012 Sep 11;24(46):6136–40. doi: 10.1002/adma.201202446 (PMC3546373; doi:10.1002/adma.201202446)
Supplement: Supplementary file 2 [file adma0024-6136-SD2.pdf]

Copyright WILEY-VCH Verlag GmbH & Co. KGaA, 69469 Weinheim, Germany, 2012.

ADVANCED MATERIALS

Supporting Information

for *Adv. Mater.*, DOI: 10.1002/adma.201202446

Dry Lithography of Large-Area, Thin-Film Organic Semiconductors Using Frozen CO_2 Resists

Matthias E. Bahlke, Hiroshi A. Mendoza, Daniel T. Ashall, Allen S. Yin, and Marc A. Baldo*

Supporting Information

Double Laser Interferometric CO_2 film thickness measurement

In order to monitor and calibrate growth of the frozen resist film as well as measure its density, an apparatus utilizing dual beam optical interfence was constructed within the PVD system. A functional schematic of this setup is shown in Figure S1. As demonstrated in references [S1–S3], reflection of light off carbon dioxide deposit growing at a constant rate exhibits interference peaks according to the following (derived from Bragg's refraction equation):

$$\frac{d\tau}{dt} \cdot \Delta t = \frac{\lambda}{2n\sqrt{1 - \frac{\sin^2\theta}{n^2}}} \tag{S1}$$

where $\frac{d\tau}{dt}$ is the growth rate, Δt is the time between peaks, λ is the wavelength of the light source, θ is the incidence angle from normal, and n is the real part of the film's refractive index. When two light sources are incident on the substrate at two different angles, we get two equations that can be rearranged to solve for the refractive index and the growth rate:

$$n^2 = \frac{\sin^2\beta - \frac{\Delta t_\alpha}{\Delta t_\beta}^2 \sin^2\alpha}{1 - \frac{\Delta t_\alpha}{\Delta t_\beta}^2} \tag{S2a}$$

$$\frac{d\tau}{dt} = \frac{1}{\Delta t_\alpha} \cdot \frac{\lambda}{2n\sqrt{1 - \frac{\sin^2\alpha}{n^2}}} \tag{S2b}$$

$$= \frac{1}{\Delta t_\beta} \cdot \frac{\lambda}{2n\sqrt{1-\frac{\sin^2\beta}{n^2}}} \tag{S2c}$$

where Δt_α and Δt_β are the periods between interference peaks from reflections with incident angle α and β.

A Radius 405-25 CDRH, 25 mW laser provides the light signal, which is reflected into the vacuum chamber through the viewport. The beam is then split into two and reflected toward the substrate near the top of the chamber. Two Newport-818 photodetectors inside the chamber receive the reflected beams and two SR830 lock-in amplifiers are used for signal acquisition.

The substrate is a piece of 2"×2" borofloat glass coated with 100 nm of aluminum to be specularly reflective but still have similar thermal properties as the OLED substrate. It is secured onto a 4"×6" substrate holder, which can be screwed into a 6"×6" cooling chuck. The cooling chuck is attached to the top of the chamber via nylon screws and nuts for thermal isolation. Liquid nitrogen is circulated through the chuck to provide cooling for the substrate holder and substrate. Both the chuck and substrate holder are machined from OFHC copper and indium foils are sandwiched between every interface to promote heat conduction.

A thermocouple mounted on the substrate holder measures 98-100 K during cooling, and we estimate the substrate to be between 102-104 K. CO_2 is flowed into the chamber via a leak valve such that during deposition the chamber pressure is raised to 1.0-3.6 x 10^{-3} Torr from 10^{-7} torr base pressure. Photodetectors yield declining oscillations with distinct, constant periods. A quartz crystal microbalance (QCMB) mounted on the substrate holder also yields roughly linear decrease in frequency during deposition, indicative of constant deposition rate.

From the growth rate derived from Equation S2 and the instantaneous mass increase derived from Sauerbrey's equation[S4], an estimate of the resist density is obtained to be

1.51±0.15 g cm^{-3} for the range of pressures in these experiments. The density versus pressure is shown in Figure S2. The refractive index is calculated to be 1.5±0.07. These quantities compare well with references [S1–S3]. Both the rate of QCMB frequency decrease and deposition rate exhibit linear relationships with pressure. From their linear fittings, we calculate that 1.51±0.15 g cm^{-3} can be used for the crystal monitor's density parameter to reliably estimate thickness growth in this apparatus. The resist growth rate is easily controlled from 3-12 nm s^{-1} using a variable leak valve and roughly 10 μm s^{-1} bleeding in CO_2 on the order of 50 sccm.

It is worth noting while the experiments yield consistent and reasonable results, they do not say much about the uniformity of the resist layer as only one incident spot on the substrate is measured at a time.

Stamp Profile

A micrograph of the SU-8 stamp used in these experiments is shown in Figure S2. A pillar has been knocked down on its side to display the tapered profile of the pillars. The edge of the fallen pillar in the lower portion of the figure is that which meets the frozen CO_2.

Supporting Figures

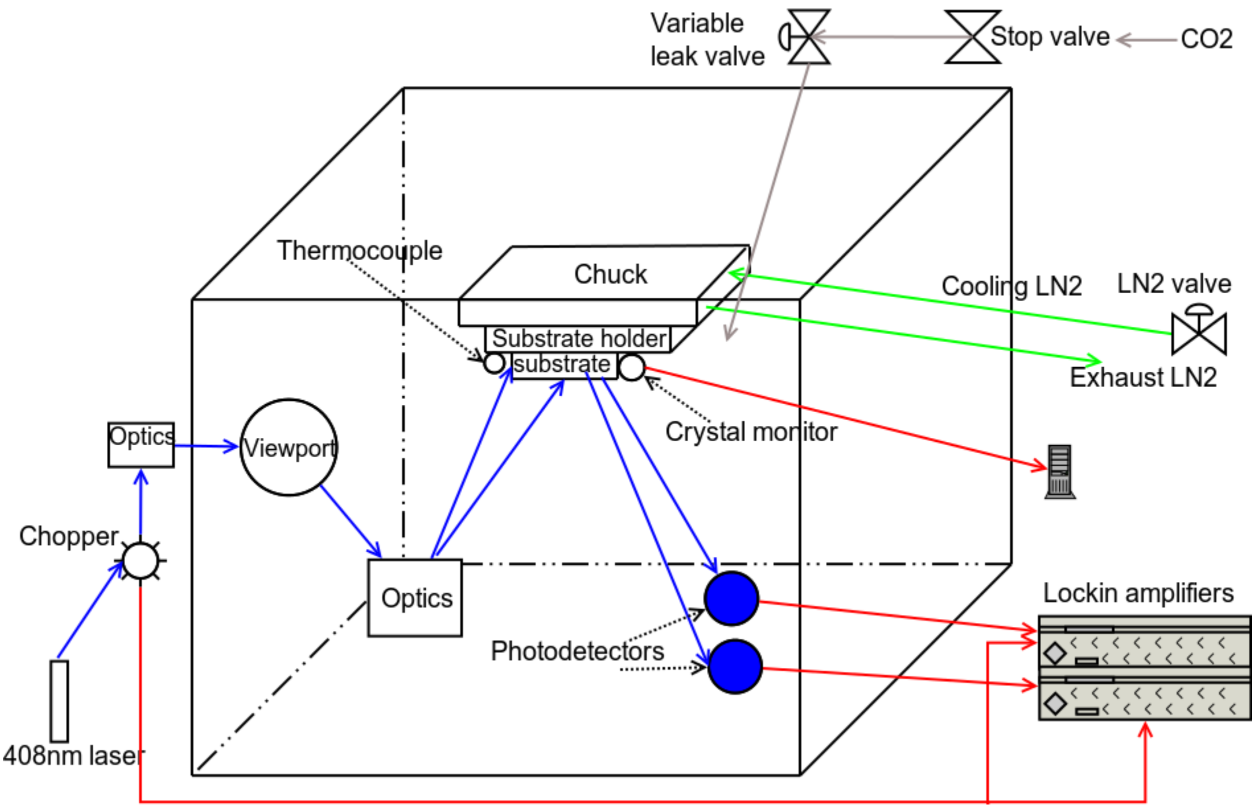

Figure S1. Functional schematic of the interferometer setup. Everything inside the box represents those installed inside the vacuum chamber; Blue lines represent light paths. Red lines represent electrical/signal paths. Green lines represent LN2 flow. Gray lines represent gas flow. Arrows indicate the direction of flow/travel.

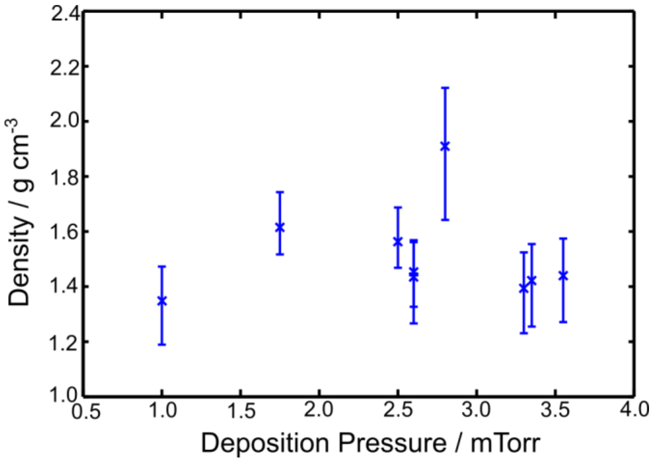

Figure S2. Density vs. Pressure as measured by double laser interferometry in our system.

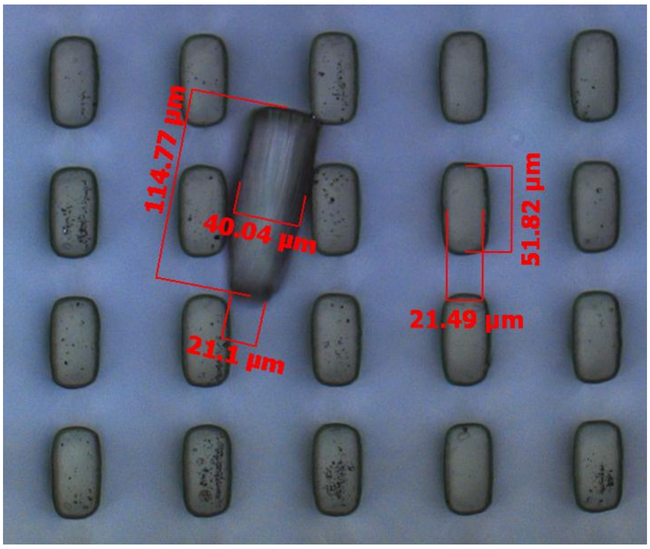

Figure S3. Micrograph of a region of the SU-8 stamp used to selectively sublime regions of the CO_2 resist. In the center of the image is a pillar that has been knocked over displaying the profile of the pillar.

CO_2 Resist Lift-off

A video capturing the lift-off of the CO_2 resist as its temperature is increased is available at the web address for this communication.

Supporting Information References

[S1] M. Á. Satorre, M. Domingo, C. Millán, R. Luna, R. Vilaplana, C. Santonja, *Planetary and Space Science* **2008**, *56*, 1748–1752.

[S2] M. Domingo, C. Millán, M. Á. Satorre, J. Cantó, SPIE, **2007**, p. 66164A–66164A–8.

[S3] K. E. Tempelmeyer, D. W. Mills, *Journal of Applied Physics* **1968**, *39*, 2968–2969.

[S4] G. Sauerbrey, *Z. Physik* **1959**, *155*, 206–222.
